# Legal Complexities of Entry, Rescue, Seizure and Disposal of Disaster-Affected Companion Animals in New Zealand

**DOI:** 10.3390/ani10091583

**Published:** 2020-09-04

**Authors:** Steve Glassey

**Affiliations:** School of Social Sciences, University of Otago, P.O. Box 56, Dunedin 9054, New Zealand; steve.glassey@postgrad.otago.ac.nz; Tel.: +64-210-278-8930

**Keywords:** animal, disaster, emergency, law, rescue, welfare, seizure, disposal

## Abstract

**Simple Summary:**

Companion animals are increasingly seen as a valued member of the family unit, and when disaster strikes their guardians often act protectively of them even at the risk to human safety. This behaviour has been observed in numerous disasters and as a result of Hurricane Katrina in 2005, the United States passed specific federal law to protect companion and service animals in a bid to acknowledge that in doing so it would also benefit the wellbeing and safety of its citizens. This article explores the effectiveness of current legislative arrangements in New Zealand with a focus on powers to seize and dispose of companion animals during and following an emergency, as well as other legal considerations for public safety. Though specific to New Zealand, the recommendations provide generic considerations that may enhance the legislative frameworks in other countries to improve both animal and human safety and wellbeing.

**Abstract:**

With the increasing societal expectation that animals are afforded greater protection in emergencies, the legal process from entering a property to rescuing a companion animal, through to how to dispose of such animals if they remain unclaimed has not been well examined in New Zealand. It is hypothesised that the legal framework for such a response is flawed. In this study, each phase of animal disaster rescue is evaluated against four key statutes that may apply in each phase, in that does any statute provide clear end-to-end provisions with clear legal authority to do so? The study found that all statutes evaluated contained flaws and that the current legal provisions are insufficient to provide clear authority for the sequential process of undertaking the rescue of animals during emergencies. A major flaw was discovered in the Civil Defence Emergency Management Act 2002, a key statute, that provided for the seizure of property and animals but omitted a procedure for the disposal of such seized things leaving them all in legal limbo. It is recommended that animal disaster laws be updated to be more animal inclusive. The method also may be applicable to assist evaluating animal disaster management legal frameworks in other countries.

## 1. Introduction

The current animal disaster legal framework in New Zealand is based primarily upon the Animal Welfare Act 1999 and Civil Defence Emergency Management Act 2002. Both of these were written prior to Hurricane Katrina (2005) which was the genesis for modern animal disaster law with legislation being swiftly passed due to lessons identified, such as the Pets Evacuation and Transportation Standards (PETS) Act 2006 [1]. According to the Fritz Institute [2], 44% of those who chose not to evacuate during this catastrophic event did so in part because they were unable to take their pets as the federal policy was to leave pets (companion animals) behind at that time. Now in the USA, the PETS Act 2006 requires federal, state and local plans to include animal rescue, evacuation, sheltering and care.

Closer to New Zealand, following the 2009 Black Saturday bushfires in Victoria, Australia, the Royal Commission looking into this disaster found that human lives were lost as a direct result of animals not being able to be evacuated and pet owners returning prematurely to their properties to save their animals [3]. Significantly, the Royal Commission also recognised animal suffering and loss as inherently undesirable outcomes in disasters (A. Best, personal communication, 2020). A crucial development that came from this inquiry was the introduction of the Victorian Emergency Animal Welfare Plan, which has been described as the most robust instrument of its kind in Australia (A. Best, personal communication, 2020). According to White, “neither animal welfare law nor emergency management law address the management of the welfare of companion animals in disaster situations (in Australia) in any comprehensive way” and that “the interests of companion animals continue to be inadequately addressed” [4]. The lack of equivalent animal-specific disaster management legislation was also observed by Taylor et al. [5].

### 1.1. Animal Welfare Emergency Management Framework

The Civil Defence and Emergency Management Act (CDEMA) 2002 yielded a robust and forward thinking piece of legislation that has served the country well for most part given its grounding in comprehensive emergency management that covers the phases of risk reduction, readiness, response and recovery [6]. The CDEMA provides high-level responsibilities, powers and functions. It also allows for the creation of regulations and orders, including the National CDEM Plan Order that provides detail on national coordination arrangements. Such Orders are made as a schedule to an Order in Council. A “Guide to the National Civil Defence Emergency Management Plan” is also published to accompany the NCDEM Plan in a less legalistic format that also has supplementary information for users to enable the intent of the plan to be achieved.

The first National Civil Defence Emergency Management (NCDEM) Plan Order (herein the NCDEM Plan), was introduced in 2005 under the CDEMA and included an animal welfare section, with local authorities recognised as providing the animal welfare function for civil defence locally. The 2005 NCDEM Plan also stated that the Ministry of Agriculture and Forestry (later becoming the Ministry for Primary Industries) was only responsible for a national level reporting capability to government (cl. 48(1)).

The current NCDEM Plan was introduced in 2015 and continued to make provisions for animal welfare, but the key change was that it placed responsibility on the Ministry for Primary Industries (MPI) to nationally and regionally “coordinate” animal welfare emergency management also. The role of local authorities in animal welfare emergency management was also diminished and the core tenets of the PETS Act had not been replicated in New Zealand law despite the experiences of 2005 (Hurricane Katrina USA), 2009 (Victorian Bushfires) or the 2010–2011 Canterbury NZ earthquakes. Seventeen years after the first NCDEM Plan, there still is no national animal emergency management plan and only a small handful of group-level animal emergency management plans exist [7].

Although the lead agency (MPI) was mandated to have an emergency management plan for its responsibilities and to take all necessary steps to ensure those functions are provided (s. 59, CDEMA), no such national plan exists with such related work being dropped in favour of developing a “strategy”, that also remains unpublished.

Since 2009, state-level animal welfare emergency plans have also been developed across Australia, including in New South Wales, Western Australia and South Australia, for pets, livestock and wildlife [8]. It would appear that New Zealand is not keeping pace with best practice for animal emergency planning within Australasia.

In addition to the CDEMA, New Zealand has other legislation that contributes to the framework that affects animal welfare emergency management. This includes the Animal Welfare Act 1999 (AWA), the Dog Control Act 1996 (DCA) and the recent Fire and Emergency New Zealand Act 2017 (FENZA). None of these statutes were developed with specific regard to animal welfare in disaster situations.

In the Animal Evac New Zealand (AENZ) report, presented to Parliament in 2019 the deficiencies in law were identified with recommendations for improvement provided [9]. These deficiencies included the legal practicalities of carrying out animal disaster rescue, from entering onto private property to how rescue animals could be rehomed if not claimed (Table 1) and colour coded each statute versus phase for legal effectiveness for the circumstance and context of animal disaster response, with red being ineffective, orange being of limited effectiveness and green being effective.

Reference to the American Bar Association’s (ABA) “Model Act Governing Standards for the Care and Disposition of Disaster Animals (2/10)” [10] is made in the matrix (Table 1) also.

The legal phases of animal disaster rescue in the matrix (Table 1) were summarised as follows:

1. Power to enter property
2. Power to enter dwellings
3. Power to rescue
4. Notification of entry or seizure
5. Disposal of animals rescued

### 1.2. Evacuation Prior to Animal Disaster Rescue

Prior to or during the animal disaster rescue phases, evacuations may occur. These evacuations may be self-initiated by property occupants, or such occupants may be instructed to evacuate voluntarily or under order. An evacuation order is mandatory and can be given by a controller or constable under s.86 of the CDEMA. However, the current Act has human-centric wording leaving animals unprotected. Section 86 requires evacuations to be necessary for the preservation of “human life” and only provides for the exclusion of “persons” and “vehicles”.

The inconsistent use of “life” and “human life” within the CDEMA creates challenges as the interpretation of “life” may extend to animals, whereas “human life” is very specific. It is assumed “life” refers to all life, but without clear definition within the Act, it remains ambiguous and open to interpretation. These discrepancies were raised as issues with government in 2010 (and 2019) and despite recent amendments to the CDEMA in 2016, these and other animal emergency management issues continue to be ignored.

The refusal of public safety officials to allow companion animals to be evacuated alongside their human families is a leading cause of evacuation failure [2,11,12,13]. The omission of animals in section 86 of the CDEMA may also imply that animals cannot be excluded from a premise or place.

The NCDEM Plan does have animal inclusive principles pertaining to evacuation planning and operations (cl.140(d)) but fails to recognise animals may require mass evacuation under clause 138 (mass evacuations), and not in the CDEMA. The NCDEM Plan requires that the primary responsibility for the welfare of animals lies with the owners or person in charge of the animals (cl.140(d)(i)); that evacuation of companion animals and disability assist dogs, occurs alongside people (cl.140(d)(ii)); and the evacuation of production and other non-companion animals is the responsibility of the owner or person in charge of the animals (cl.140(d)(iii)).

The NCDEM Plan also requires that evacuation planning is collaborative involving all stakeholders and includes where possible, consultation with affected communities (cl.140(c)). It would appear from recent events such as the Edgecumbe Flood (2017) and Nelson Fires (2019), that companion animals have not been consistently evacuated alongside people, and that evacuation plans involving animal welfare stakeholders or the community had not been developed as expected [7], highlighting that the NCDEM Plan may not be creating the effects intended.

## 2. Materials and Methods

Each legal phase of animal disaster rescue was evaluated for effectiveness against the four key statutes affecting animal welfare emergency management, those being the CDEM Act 2002, Animal Welfare Act 1999, Dog Control Act 1996 and the Fire and Emergency New Zealand Act 2017. The question was: did the statute provide clear lawful authority for each animal disaster rescue phase? This study goes beyond the cursory evaluation as provided in the 2019 Animal Evacuation New Zealand (AENZ) report [9], to provide more detailed legal commentary. In effect, this study attempts to provide a chronological walk-through of a typical response requiring public safety responders to search for and rescue companion animals during and following a declared state of emergency, and the management of rescued or displaced animals where no owner has come forward.

This study excludes disaster-related issues observed relating to the protection of disability assistance dogs; rental accommodation shortages following disasters; complexities of animal registration and microchipping databases; ethical requirements to protect animals involved in research; animal control jurisdictions during response; management of deceased companion animals; or whether the existing lead agency is appropriate to lead the animal welfare emergency management function. These issues are primarily discussed in the AENZ report [9]; however, they will benefit from further research.

## 3. Results

### 3.1. Power to Enter on to Property

Under the Animal Welfare Act 1999 (AWA), an Animal Welfare Inspector (which includes a constable, herein Inspector) may enter a property to inspect an animal under section 127. An authorised person under section 42 of the Fire and Emergency New Zealand Act 2017 (FENZA) may also enter a property to protect life and property. The argument whether animals are “life” or “property” are not considered in this study, but “life” is assumed to include animal life. The Dog Control Act 1996 (DCA) allows Dog Control Officers to enter property to check the conditions of dogs under section 15(1)(c); however, Dog Control Officers do not have the power to enter a property to check on other species of animals which is limiting in an emergency situation. In most cases, Dog Control Officers are employees or contractors to the local authority. Under the Local Government Act 2002 (LGA), such officers may also have powers under s.173 to enter occupied land or buildings in a sudden emergency that is causing or is likely to cause loss of life or damage to property. Finally, the CDEMA allows a constable or any person authorised by a controller to enter a property, but only during a declared state of emergency (s.66 or s.68) meaning no such powers are available in the lead-up to a declaration which often is hours after the onset of sudden emergencies. In this animal disaster rescue phase, it appears that only the AWA and FENZA provide clear and existing powers to enter onto a property regardless of species at risk or whether there is a state of emergency declared or not.

### 3.2. Power to Enter a Dwelling

After making entry on to a property during an emergency, it is a common requirement for animal disaster rescuers to enter dwellings to ensure animals have not been left behind. The legal definition of a dwelling during an emergency is also open to interpretation as can a house be occupied as a dwelling if it is subject to mandatory evacuation, and is it still a dwelling where it is so damaged that it is unable to be habited? These two questions require further legal analysis; however, for the purposes of this study, we assumed that, under a conservative approach, dwellings retain their legal status regardless of the circumstance. This is important as a dwelling is often sacrosanct under law to protect the rights of occupants and it should be noted dwellings can include any building or structure for human habitation and may include motorhomes and tents. Both the AWA and DCA require the person exercising powers of entry to have a search warrant (subpart 3, Search and Surveillance Act 2012) to enter a dwelling, even in a disaster as the CDEMA does not affect the powers, functions or duties of other acts (s.6). It is not practical for emergencies involving multiple dwellings to seek a search warrant for each property making both Acts ineffective in animal disaster rescue. The CDEMA, however, does provide the power to enter properties and buildings (s.87), as it does not mention any special caveats for dwellings, but such power to enter any building regardless of its purpose (such as being used for human habitation) is only available during a declared state of emergency. Whether a state of emergency is in effect or not, those authorised under the FENZA may enter any land, building or structure (s.42(2)(a)); may use force to do so (s.42(2)(b)); and, such authorised persons are protected from liability in doing so (s.161). Similar protections for reasonable forced entry and other damages are made under the AWA (s.158), DCA (s.74) and CDEMA (s.110). It should also be noted that *marae* (indigenous land that is afforded special government status) has the same protections as a dwelling under the AWA, meaning that entry on to this land and any of its buildings by an inspector or constable requires a search warrant. In this animal disaster rescue phase, it would appear only the FENZA provides clear and existing authority to rescue animals from dwellings regardless of a state of emergency being declared.

### 3.3. Power to Rescue

The rescue of animals is important to human safety. The academic consensus that in an emergency, saving animals in effect saves human lives, is a fundamental philosophy to contemporary emergency management doctrine. In New Zealand, there have been frequent examples of people losing their lives in an attempt to rescue their companion animals [14,15] and similar occurrences are common overseas too. In 2017, a woman who was refused entry to the cordoned off township of Edgecumbe following flooding while trying to get to her horse, defied the cordon and secretly swam across the flooded river to successfully get her horse to safety [9].

During the animal disaster rescue phase of having already entered a property including a dwelling, when an animal is located and it is in need of being rescued, most of the statues being evaluated provide for such powers. The CDEMA provides for persons under the direction of a controller or constable to seize things, including animals to limit the extent of the emergency (s.92). An Inspector (including a constable) may take an animal into possession where they believe it to be at risk from imminent harm (s.127(5)(c)). The CDEMA also provides for the rescue of people (s.85(1)(b)), but not animals.

Under the FENZA, it is assumed under s.40(b) that an animal may be rescued as part of taking “whatever action is necessary to save lives and property in danger”. The assumption that animal rescue is within the scope of an authorised person under the act is reflected in the statutory additional assistance function of FENZ to perform rescues involving animals (s.12(3)).

The only statute that is not effective in this phase is the DCA which is limited to situations involving dogs, and that only where dogs have limited access to food, water or shelter may they be seized (s.15(1)(c)). This means under the DCA, where a dog is found on a property in need of evacuation (and consent of the owner is not available), and that dog already has food, water and shelter, it may be considered unlawful for the dog to be seized. It could be argued that if a property is about to become flooded or the area is evacuated and persons are not permitted to enter, that this creates a situation where the dog will have limited access to food or water and therefore provides grounds for seizure (s.15(1)(c)). If a dog is in a public space or on private property where such property owners give consent, the dog can be impounded by a dog control officer. In situations where dogs are roaming off their property during an emergency, there are provisions for seizing (impounding) the dog under the DCA. The effectiveness of the DCA to seize dogs for the purpose of protecting them during an emergency is heavily influenced by situational factors. On this basis, the DCA is not effective in providing adequate protection for dogs in emergencies.

### 3.4. Power to Requisition to Assistant Animal Rescue

To carry out activities for the preservation of human life under the CDEMA, such as rescue activities, the Act provides for the requisitioning of equipment (s.90). This could include the requisitioning of boats to rescue people, but the Act unfortunately is inconsistent through its sections with some powers specific to preservation of “life” and others “human life”. By limiting emergency powers such as requisitioning to only “human life”, rather than having powers to requisition to preserve “life” that would then include animals, the Act in its current form may inadvertently put human life at risk.

For mass animal rescues during disasters such as those from intensive farming facilities and laboratories, specialist equipment and heavy machinery may be needed. The inability of public safety officials to be able to carry out specialist or logistically complex animal rescue operations may force some to defy official advice and put themselves in harm’s way as seen in numerous events such as the 1996 Weyauwega train derailment [11], Buckeye Farm disaster in 2000 [11], Fukushima nuclear incident in 2011 [16] and the Edgecumbe Flood in 2017 [9].

### 3.5. Notice of Entry or Seizure

Where statutes are focused on law enforcement such as the AWA and DCA, rather than public safety such as the CDEMA and FENZA, there are requirements for inspectors, constables and dog control officers to leave a notice of entry, and where things are seized, further written record of what was taken from the property or person. Such requirements are part of ensuring checks and balances are in effect when enforcement powers are being exercised.

However, in an emergency situation and especially those situations where multiple properties require entry, the issuing of such notices may not be practical. For example, following the evacuation of the township of Edgecumbe in 2017 following a major flood as a result of a sudden flood bank protection failure [17], over 600 properties were required to be searched for abandoned animals. This became the largest companion animal rescue operation in New Zealand’s history and if a physical notice had to be issued to each property this would have been problematic as there were not enough notices (as prescribed in s.129) available; the writing up on a notice for each property would have delayed the rescue operation; and, the use of the prescribed forms on paper were not compatible for use in flood conditions. Powers to enter property, dwellings and seize were provided for under delegation by the CDEMA for this event.

In most day to day cases where inspectors or dog control officers need to leave a notice of entry under their respective statues, this is done usually by affixing the notice to the front door of the dwelling or being placed in the letterbox. In overseas events, floods have been so high that only rooftops are exposed leaving this the only place to affix a notice of entry which is somewhat not useful once property owners return after floodwaters recede. In other disasters, such as earthquakes, structures may be left in ruins, again leaving the requirement to affix legal notices an issue. Though not compliant with the requirement of s.129 of the AWA, it is common for rescue teams, both human and animal, to mark properties searched with disaster search markings (often with spray paint) that may indicate how many people or animals have been rescued, alive or deceased [18] and the CDEMA provides for the power to affix such markings (s.92) and without liability for damage in doing so (s.110).

Where entry and removal of animals is undertaken under the CDEMA or FENZA, no such notice of entry is required. The CDEMA continues to have limitations in that it only provides such powers during a state of emergency. The FENZA would appear to be the only statute providing clear and existing uncomplicated powers to rescue animals from properties during an emergency.

### 3.6. Disposal of Animals Rescued

Where animals have been rescued during an emergency under the AWA, CDEMA, DCA or FENZA, and the owner or person in charge has failed to reclaim them, the animals need to then be disposed of. The term “disposed of” is a legal term within the AWA and DCA and should not be assumed to be mean the animal is destroyed. Under the AWA, disposal of animals could include selling, adoption, auction, sale, transfer (to another animal organisation) or euthanasia. Currently, in New Zealand, only the Royal New Zealand Society for the Prevention of Cruelty to Animals (SPCA) is an “approved organisation” under section 121 of the AWA. Approved Organisations have the powers to enforce the AWA and also receive and dispose of animals presented to it, such as those animals that are abandoned, lost or displaced.

Once an animal comes into the custody of the SPCA as an approved organisation under the AWA, the SPCA can re-home the animal or otherwise dispose of it after 7 days pursuant to section 141(1A) if the owner does not claim the animal, unless the animal is taken into possession by an inspector under section 127. The disposal provision under section 141(1A) is applicable where, for example, a member of the public delivers an animal they have found (in an emergency or not) into the custody of an approved organisation. Where an animal has been taken into possession by an inspector under section 127, unless the inspector returns the animal by agreement or surrendered by the owner (transferring ownership to the approved organisation), only the district court can order the disposal if it deems it appropriate after an application is made by the inspector (s.136A). This process can take months and, therefore, it creates a significant disadvantage of using the AWA in an emergency to rescue (take into possession) an animal, notwithstanding the complexity of notice of entry requirements.

The NCDEM Plan, however, places the local authority as the organisation responsible for accommodation of companion animals, yet they (and all other animal-related organisations in New Zealand other than the SPCA) do not have the legal authority to re-home unclaimed animals other than dogs (as local authority powers for disposal only extend to stray dogs found at large under the DCA) and they have no powers for holding or disposing of displaced companion animals such as cats, rabbits and birds.

A major flaw in the CDEMA is that it does not provide for the disposal of seized items except for destruction, which would have to be done while a state of emergency is still in effect. This means for animals seized under the CDEMA during a state of emergency, once the state of emergency has been lifted or expires, such animals have no legal process for their disposal if unclaimed. This creates the risk where if they animal is euthanised that no such authority exists, and where the animal is re-homed, the lack of legal process for ownership transfer may lead to animal custody disputes as experienced after Hurricane Katrina.

After overseas experiences including Hurricane Katrina, the American Bar Association created a model act for states to adopt to address the ownership, temporary holding, transferring and disposal of animals during and following a disaster [10]. Their recommendation was that during a declared disaster, that the holding period was set at 30 days to allow for displaced owners to claim their animals; and that animals could not be transferred out of state without approval of the State Veterinarian [10]. Thousands of animals were evacuated and transported across the United States following Hurricane Katrina, never to be reunited with their original families again and this prompted legal reforms [19]. The model act also ensured that animals that were unable to be reunited could be legally re-homed with ownership being transferred.

Where animals have been rescued and removed from a property under FENZA, there is no legal procedure for the disposal of such animals that are unclaimed. Animals rescued under FENZA in such circumstances could be transferred to an approved organisation which assumes custody of it, and then after following requirements to return the animal to its owner, it could then be disposed of after seven days. This leaves only the provisions of disposal under section 141(1A) to give effect to re-homing (or otherwise) of unclaimed animals and this power only extends currently to the SPCA (as the only approved organisation under the AWA) which is not even responsible for the care, transport and accommodation of disaster-affected companion animals as specified in the NCDEM Plan.

## 4. Discussion

After consideration of the above factors, none of New Zealand’s relevant statutory regimes provide a clear and effective end-to-end legal process for animal disaster rescue, from entry on to property to make a rescue, to the disposal of such animals that remain unclaimed. Unless otherwise specified, laws need to provide continuity of legal process, and in the context of this study, emergency responders have no clear or effective process to follow, creating risk for themselves and their organisations. The least complex process for animal disaster rescue was under the FENZA; however, this is based on some assumptions in that it is implied that animals can be removed from a property or dwelling as part of the authorised persons power to “take whatever action is necessary to save lives and property in danger” (s.40(b)), and the definition of lives extends to animal lives. FENZA is also limited as it has no disposal provisions where things are seized or taken into possession if that is the action chosen by the authorised person. This leaves an assumption that the disposal arrangements are reliant on the animal being delivered into the custody of an approved organisation and the default disposal provisions of section 141(1A) being applied where no owner claims the animal, acknowledging the seven-day requirement under the section is insufficient in the aftermath of a disaster according to the American Bar Association. It is clear that the animal disaster rescue laws in New Zealand are not fit for purpose and have significant limitations. Though there has been some criticism of the PETS Act 2006 being described as “no carrot and no stick” [1] and having some deficiencies [20,21], the passage of this law has been labelled as “effective” [22] and having positively influenced the culture within emergency management to afford greater protection to animals during and following disaster [1].

To address these limitations, several recommendations are offered.

### Recommendations

1. Mandating within the National CDEM Plan:
  - The development, maintenance, resourcing and exercising of animal welfare emergency management plans, both at the national and regional level.
2. Amending the CDEMA to be more animal inclusive, by:
  - Replacing reference to protective measures (part 5) from “human life” to “life” or replacing with “human and animal life” across the Act to ensure such measures can be applied to animals too.
  - That the term “animal” is included in the interpretation (s.4), with “animals” being defined as per the meaning given in s.2 of the AWA.
  - Amending the CDEMA to include a section on disposing of things seized, with special attention to animals including a 30-day holding period, transfer of ownership for unclaimed animals and for such provisions to continue once a state of emergency has been lifted.
  - Amending the CDEMA to specifically provide for emergency powers under section 85 to provide for the evacuation, rescue, transport, accommodation and essential needs of animals.
3. Amending the CDEMA to specifically provide the power clarify markings under section 92 to include the definition of “marking” as per the meaning given in the AWA (s.2) to cover implanting of animals with microchips.
4. For the purposes of consolidation, consideration should be given to a specific act or regulation made under the CDEMA, such as that of the Pet Evacuation and Transportation Standards Act 2006 set in the United States that ensures planning, funding, public transportation and rescue capability emergency arrangements are in place for companion animals.
5. The issues identified in the AENZ report [9] that have not been addressed in this study require further attention.

## 5. Conclusions

There is considerable evidence that substantiates the protective nature of humans towards animals, in particular companion animals. Well respected disaster management scholar Erik Auf der Heide stated that emergency planning should be based on “normal behaviour” not “correct behaviour” [23]: in effect we should plan on the basis on how humans will likely react, not how we want them to react. On this basis, emergency managers and law makers need to place greater focus on ensuring that animals, in particular companion animals are acknowledged as intrinsically linked to people. To achieve improved evacuation compliance and public confidence in response coordination, the welfare of animals during emergencies needs to be a core function and a priority of the response. To enable this change and designate accountability, New Zealand needs to heed the lessons of Hurricane Katrina, the Black Saturday Victorian bush fires and the Edgecumbe Floods, and give urgency to strengthening the animal emergency management laws with amendments to the relevant acts or the passage of specific regulations to reflect international best practice and meet the expectations of its citizens.

## Figures and Tables

**Table 1:** Legal phases of animal disaster rescue (revised) [9].

Acts	Power Bestowed upon	Power to Enter onto Property	Power to Enter Dwelling	Power to Rescue Animals	Notice of Entry Required	Disposal of Animals Rescued	Disposal of Animals Presented	Disposal Meets ABA Model Law (30-Day Hold)
CDEMA 2002	Controller or any Constable	Conditional to declared state of emergency only [s.87]	Conditional to declared state of emergency only [s.87]	Yes [s.92]	No	No provisions for things seized	No provisions	No
AWA 1999	Animal Welfare Inspector, including Any Constable	Yes, power to inspect any animal. [s.127]	No, unless Search Warrant issued. [s.127(3)]	Yes [s.127(5)(b/c)]	Yes [s.129]	Where taken into possession, by court order if not returned. [s.127(6)]	After 7 days excluding stock [s.141]	No
DCA 1996	Dog Control Officer or Ranger, or any constable	Conditional to situations involving dogs [s.15(1)(c)]	No, unless Search Warrant issued.	If limited access to food, water or shelter [s.15(1)(c)]	Yes [s.15(3)]	After 7 days from notice being issued to owner [s.71A]	Dogs only after 7 days [s.69]	No
FENZA 2017	Authorised person under Act	Yes, to protect life or property [s.42]	Yes, to protect life or property [s.42]	Yes, implied by Act. [s.40(b)]	No	No provisions but may transfer to AO/TLA as not seized.	No Provisions	No
